# A Case of Hyperkalemia Induced by Kratom (Mitragyna speciosa)

**DOI:** 10.7759/cureus.24036

**Published:** 2022-04-11

**Authors:** Aldo Torres-Ortiz, Said Al Zein, Muhannad Alqudsi

**Affiliations:** 1 Department of Internal Medicine, Ochsner Medical Center, New Orleans, USA; 2 Department of Medicine, University of Pittsburgh, Pittsburgh, USA; 3 Department of Nephrology, The University of Queensland Ochsner Clinical School, New Orleans, USA

**Keywords:** substance use, adverse effects, toxicity, herbal product, hyperkalemia, mitragyna speciosa, kratom

## Abstract

Kratom (*Mitragyna speciosa*), a tree found abundantly in Southeast Asia, has been used for centuries because of its opioid-like properties. In the last 20 years, it has gained popularity in the United States of America due to its easy availability and effects on pain control. However, different types of toxicity from kratom use have been reported in the literature. Here, we present a case of kratom-induced hyperkalemia in a 61-year-old patient with no significant past medical history. His laboratory work-up excluded other etiologies, and his potassium level eventually normalized after the discontinuation of kratom. Although reasonable data exist on kratom effects on the nervous and cardiovascular systems, the magnitude of its effect on potassium homeostasis and whether it is kidney mediated or not is not well recognized.

## Introduction

Kratom (*Mitragyna speciosa*) is a tree that grows in countries of Southeast Asia such as Thailand, Malaysia, Philippines, Myanmar (Burma), and New Guinea, as well as parts of Africa. The dried leaves and small stems of kratom have been used over centuries because of its analgesic properties. Other names of kratom are ketum, biakbiak, maeng da, thang, thom, and kakuam [1]. Kratom was initially used in Malaysia and Thailand in the 18th century as a stimulant to increase work efficiency [2]. In 1940s, it was used during the Greater East Asia War as a cheaper alternative to opioids. This led to the development of the Kratom Control Act in Thailand in an effort to control its use and regain control of the opioid market [3].

In the United States of America (USA), kratom use is relatively recent, with the first reports dating from the early 2000s. Mitragynine, the active component of kratom, shows agonistic activity at opioid receptors that can lead to dependence and addiction. Hydroxymitragynine, which is a minor component, is thought to have higher analgesic properties than morphine [4]. Kratom is prepared in powder, liquid, tablets, or capsule formulations. Toxic effects of Kratom such as agitation, tachycardia, drowsiness, and vomiting have been reported in the literature. In more severe cases, seizures, respiratory depression, cardiac arrhythmias or arrest, and coma might occur [4]. However, little is known regarding its direct effects on the kidneys. To our knowledge, this is the first report of kratom-induced hyperkalemia.

## Case presentation

A 61-year-old male with a history of degenerative lumbar disc disease and hyperlipidemia was referred to our nephrology clinic due to unexplained persistent hyperkalemia. The patient was asymptomatic. He only took rosuvastatin and denied nonsteroidal anti-inflammatory drug (NSAID) or antibiotic use, smoking, alcohol intake and illicit drug abuse. His family history and physical exam were unremarkable. His blood pressure was persistently normal around 125/75 mm Hg during all office visits. Laboratory work-up from the referring clinic two months prior to our visit revealed a serum potassium level of 5.7, 5.6 and 5.8 mmol/L with one- and two-week intervals, respectively, with confirmed absence of sample hemolysis. A repeated serum potassium in our clinic was 5.3 mmol/L after the patient was on strict low potassium diet. His other chemistry tests were normal except for a mild increase in liver enzymes (Table 1). His white blood cells and platelets were normal and his kidney ultrasound was also normal.

**Table 1:** Serum chemistry and urine laboratory values for our patient ALT: alanine aminotransferase, AST: aspartate aminotransferase, BUN: blood urea nitrogen, CPK: creatine phosphokinase, eGFR: estimated glomerular filtration rate, TSH: thyroid-stimulating hormone

Test	Value	Reference range
Serum		
Sodium	141	136-145 mmol/L
Potassium	5.7	3.5-5.1 mmol/L
Chloride	104	95-110 mmol/L
CO_2_	33	23-29 mmol/L
Creatinine	0.8	0.5-1.4 mg/dL
eGFR	>60	>60 mL/min
BUN	18	6-20 mg/dL
CPK	73	20-200 U/L
TSH	1.5	0.400-4.000 uIU/mL
Cortisol	13.6	4.46-22.7 ug/dL
Aldosterone	3.6	0-30 ng/dL
Renin	0.88	0.167-5.380 ng/mL/h
AST	47	<39 units/L
ALT	73	30-65 units/L
Urine analysis		
Potassium	14	12-129 mmol/L
Creatinine	16	20-320 mg/dL
Protein	<7	0-15 mg/dL
pH	7	5.0-8.0
Specific gravity	1.02	1.010-1.025

**Figure 1:**
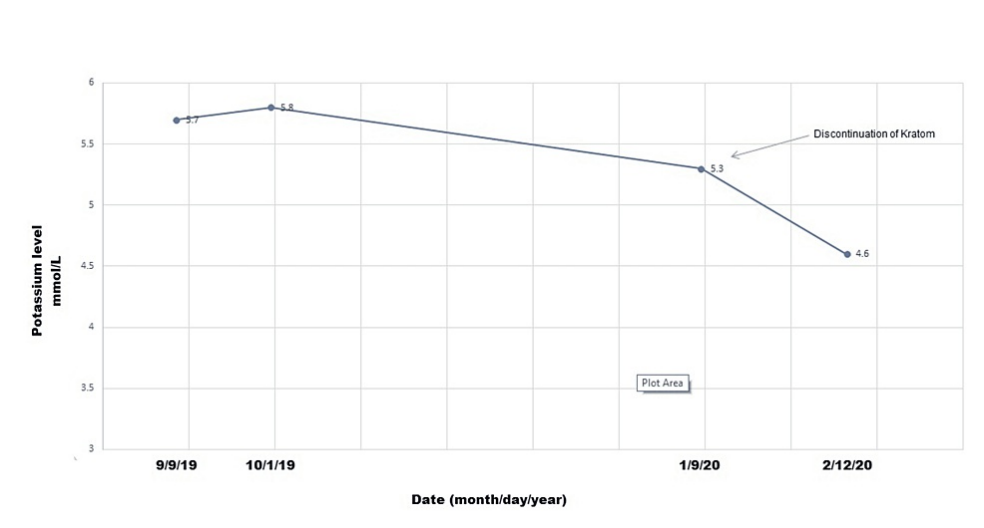
Normalization of the serum potassium level after stopping kratom

Upon re-evaluation, the patient reported using kratom for recreational purposes almost on a daily basis for the past four months. One month after kratom discontinuation, his blood chemistry revealed normalization of the potassium level (Figure 1).

## Discussion

As kratom can be obtained over-the-counter in the USA, its popularity has recently increased with an estimated 10-15 million consumers. It is typically used for pain relief or for recreational purposes due to its effects on mood (euphoria), sociability, and productivity. It is also used to self-treat opioid use disorder or other substance abuse disorders [5].

The Food and Drug Administration (FDA) has not approved kratom for any medical use. Despite the awareness of its potential toxicity and being classified as a “drug and chemical of concern,” kratom is not considered a controlled substance by the Drug Enforcement Administration (DEA). Nevertheless, there may be some state regulations or prohibitions against the possession and use of kratom. According to the National Poison Data System (NPDS), between 2011 and 2017, there were 1807 reports of kratom exposures in the USA, mostly between 2016 and 2017. About one-third of them resulted in admission to health care facilities and 51.9% resulted in serious medical outcomes [6]. The abundance of case reports in the literature has outlined the toxic effects of kratom. Withdrawals, neonatal abstinence syndrome, hypothyroidism, hypogonadism, liver toxicity, seizures, coma, posterior reversible encephalopathy syndrome (PRES), acute respiratory distress syndrome (ARDS), overdose toxidrome and fatalities have all been reported [7].

Levels of mitragynine and two of its metabolites (5-desmethylmitragynine and 17-desmethyldihydromitragynine) can be qualitatively monitored in the urine [8]. In our patient, however, these were not measured. Data on kratom effects on potassium homeostasis and kidneys are scarce in the literature. A study in rats who received *Mitragyna speciosa* for 28 consecutive days revealed an elevation in serum creatinine that was statistically significant compared to controls [9]. Another study in rats revealed nephrotoxicity only upon exposure to high doses of the drug [10]. Potassium was not studied in these animal models. Liver toxicity is one of the most common serious side effects [7]. The elevation of liver enzymes in our patient was also attributed to the consumption of kratom, which might further support the etiology of elevated potassium levels.

To our knowledge, this is the first case report of kratom-induced hyperkalemia. Our patient had normal kidney function and no significant comorbidities, and was not on medications that cause hyperkalemia. His blood samples were not hemolyzed. His serum potassium level improved slightly on a strict low-potassium diet as instructed before his presentation to our clinic. Serum potassium normalized after the discontinuation of kratom. The aforementioned presentation makes us postulate an unrealized effect of kratom on potassium homeostasis through renal or extra renal handling.

Potassium is mainly an intracellular electrolyte. The maintenance of normal serum concentration of 3.5-5.0 mmol/L is important to avoid disturbances in the resting potential of the cellular membranes that can lead to serious manifestations such as fatal arrhythmias. The kidneys are the main organs responsible for potassium homeostasis. A typical Western diet contains about 50-100 mEq of potassium per day, of which 90% is secreted in the urine. The distal nephron and collecting duct are mainly responsible for potassium excretion. Principal cells mediate potassium excretion via the basal membrane Na+-K+- ATPase pump that creates a concentration gradient promoting both potassium secretion to the tubular lumen and sodium reabsorption to blood stream. Mineralocorticoids as well as distal sodium delivery play an important role in this mechanism. The renal outer medullary potassium channels (ROMK) and big K+ channels, also called maxi K+ channels, are the main channels that regulate potassium in the distal nephron and collecting duct. With-no-lysine kinases (WNKs) are proteins that regulate many important channels including the ROMK channels [11]. In hyperkalemia, the normal response of kidney is to excrete higher amounts of potassium. This can be calculated with the trans-tubular potassium gradient (TTKG), which is derived from the urine/plasma potassium ratio divided by the urine/plasma osmolality ratio. This tool provides an assessment of the trans-tubular potassium concentration gradient in the distal nephron where potassium is secreted, which reflects the mineralocorticoid activity. However, this applies only when urine is not hypotonic and distal nephron sodium delivery is not a limiting factor for potassium secretion. Based on this and other studies, TTKG has been deemed to be not reliable in determining hyperkalemia etiologies [12]. Spot urine potassium to creatinine ratio has been proposed as an alternative, where a value of less than 200 mEq/g indicates a renal defect in potassium excretion [13]. In our patient, the ratio was 87 mEq/g, pointing towards impaired renal excretion of potassium.

The effects of kratom on potassium channel disturbances have been studied in vitro. It is demonstrated that *Mitragyna* can block the human ether-a-go-go-related gene (hERG) potassium channels in cultured bovine cells. The hERG encodes the cardiac delayed rectifier rapid potassium current (IKr) that is a determinant of the duration of ventricular action potentials and QT interval. Through the inhibition of this potassium channel, *Mitragyna* likely exerts part of its toxic effects on the heart [14]. To our knowledge, no studies have been conducted on the effects of kratom at the kidney level, and therefore, the mechanism by which *Mitragyna* can lead to hyperkalemia is yet to be elucidated.

## Conclusions

To our knowledge, this is the first case reported in the literature that shows an association between *Mitragyna speciosa* (kratom) and hyperkalemia, an effect that is potentially critical and life-threatening. In the USA, the number of people using this over-the-counter herbal product continues to rise annually, which necessitates monitoring and potential regulation. This case will encourage further studies and insights into the exact mechanism of kratom-induced hyperkalemia, which in turn may deepen the understanding of potassium renal physiology.
